# A Practical Treatise on the Diseases of Children

**Published:** 1847-06

**Authors:** 


### REVIEWS.

---

Art. IV.—*A Practical Treatise on the Diseases of Children.* By D. Francis Condie, M.D., Secretary of the College of Physicians; Member of the American Philosophical Society; Honorary Member of the Philadelphia Medical Society, etc. Second edition, revised and augmented. Philadelphia: Lea and Blanchard: 1847. 658 pages.

Our medical literature comprises a fair proportion of works devoted to the diseases of children. Dewees led the way in that department; he was followed by Eberle; then came the volume the title of which is given above; and a fourth treatise was added, two years since, by Dr. Stewart, of New York. When Dr. Condie's work first appeared, we presented to our readers an extended notice of many of its interesting chapters; and as the appearance of a new edition affords a fit occasion for renewing our notice, we propose now to give an analysis of several portions not embraced in our former article. It is but just to the author to state that, in preparing his new edition, much of his treatise has been rewritten, that many additions have been made to it, while every part has been subjected to a careful revision. It embraces the results of his long experience, and the fruits of much reading on the subject.

The first chapters of the work are devoted to the *hygienic management of children.* Temperature is shown to exercise a powerful influence upon the health of the young—cold to be a prolific source of disease at that tender age. After reciting many well known facts on this subject, the author proceeds—

"From the foregoing facts, it must be evident that to maintain a sufficient degree of warmth in the air of the apartments occupied by children, is indispensable not only to their comfort, but for the preservation of their health and lives. It will not do, however, for parents or nurses to judge of the temperature required for the wellbeing of an infant by their own sensations, for what may be sufficiently comfortable to them may be destructive to the latter; nor should they suppose that because no alarming symptoms supervene immediately after the exposuie of children to cold, their constitution does not suffer: uneasiness, at first slight, is by a repetition of the cause almost invariably converted into serious disease. Thus inflammation of the throat, air passages or lungs, more or less severe, or a predisposition to incurable affections of these and and other parts, is in infants and young children often the result of exposure to a degree of cold from which dangerous consequences are least suspected to ensue.

"Infants and children of a feeble constitution, or in whom from any cause the powers of life have been depressed, should especially be guarded from exposure to the external air during cold or damp weather. Important as the enjoyment of the fresh air is to the health and comfort of infants, the practice of carrying them abroad in cold weather, under the idea of confirming their strength, or rendering them hardy, is not less cruel than absurd. A child of sufficient age and vigor to enable its system to react promptly under the depressing influence of the cold, and by an increased evolution of heat to maintain its temperature, may, it is true, sustain with impunity or even derive advantage from exposure for a short period to a moderate degree of cold. But in all others, so far from an increase of strength and vigor, or the ability to endure without injury sudden vicissitudes of temperature, being acquired by their exposure to cold, either in or out of doors, the very opposite effect will ensue, if an attack of severe disease be not immediately produced.

"While urging the importance of a due degree of external heat to the health and comfort of infants, and the necessity of carefully protecting them from exposure to even slight degrees of cold, we would not wish to be understood as recommending that the air of the rooms they occupy should be kept at a high degree of temperature. To subject children of any age constantly to an overheated atmosphere is highly improper. From the excessive stimulation thus produced, and the profuse perspiration in which their bodies are almost constantly bathed, especially during sleep, they soon become relaxed and enfeebled—their nervous system, at the same time, acquires an undue degree of irritability; every trifling vicissitude of temperature causes them to suffer, and they become liable to attacks of severe disease from the slightest causes.

A temperature of from 68° to 70° of Fahrenheit, is that best adapted to the nursery—a less degree of heat would not be prudent for very young children, and even those more advanced in age will scarcely tolerate, with perfect impunity, a much lower temperature. It is to be recollected that, notwithstanding a robust and healthy child, when a few years old, will suffer no injury from a dry and cold atmosphere, while engaged in active exercise—even out of doors—yet, when at rest, within doors, its health and comfort will be best promoted by the air of the room being sufficiently warm to prevent the least sense of chilliness.

The injurious consequences we have pointed out as resulting from constant exposure to too high a degree of artificial heat, will indicate the propriety of protecting children, as much as possible, from the intense heat which usually prevails during the summer months, especially in the cities of our southern and middle states, by a free ventilation of the apartments they occupy, and by frequent exposure to the fresh air, in open and shady situations. The deleterious effects of the heated and confined air of a large city upon the health of children may, in a great measure, be counteracted by these means, as well as by daily rides into the surrounding country, or by excursions upon the water; the means for which, in most of our larger cities, is placed within the reach of all, by the numerous steamboats that depart for short trips, at almost every hour.

It is a popular prejudice, that *dirt* is favorable to health—that children not the most cleanly are the most healthy; and we suspect there is some ground for the prejudice. It is healthy, active, spirited children who cannot easily be kept out of the dirt; they are dirty because they are healthy; not healthy because they are dirty. Dr. Condie insists upon cleanliness, and every physiologist will unite with him in the precepts he lays down on this point. A vigorous child may thrive in the midst of pollution, but how many delicate infants are stifled, in the first months of their existence, by bad air, and filthy apartments and foul clothing, no human calculation can determine. We are no hydropathists; but we are free to avow that we are ardent advocates for water, warm and cold; we subscribe to all the following:

"During the entire period of infancy, the whole surface of the body should be washed in warm or tepid water every morning—and during the day, such portions of it as may become soiled by the natural evacuations or from any other cause. Nor should the maintenance of personal cleanliness, by similar means, be neglected after the child has passed beyond the term of infancy. The daily use of the bath, and more frequent ablutions of the face, hands and feet, should still be enjoined, and any neglect in regard to them prevented by a careful surveillance on the part of the parents or guardians. Frequent bathing in tepid water, indepently of its removing from the surface every source of impurity, benefits the health of the child by promoting the functions of the skin, and encouraging the free and regular circulation of the blood through its numerous vessels, securing thus the regular growth and full developement of every portion of the body.

"'I consider bathing,' remarks Struve, 'as the grand arcanum of supporting health, on which account, during infancy, it ought to be regarded as one of those sacred maternal duties, the performance of which should on no account be neglected for a single day.'

"The time during which the child should remain in the bath will vary according to its age. For the first month after birth the immersion should not continue longer than three or four minutes—the time being gradually prolonged as the child advances in age; continuance in the bath beyond ten or fifteen minutes is, however, unnecessary and scarcely prudent at any period of childhood. Children should not be permitted to enter the bath when in a state of profuse perspiration, nor for some hours after a meal.

"Intimately connected with the subject of cleanliness is a proper attention to the hair. In early infancy, all that is necessary is to subject the head, in common with every other part of the surface of the body, to daily ablutions in warm water; as the child increases in age, and the hair begins to grow, a little soap may be added to the water with which the head is washed, and the hair should be repeatedly but gently cleansed with a soft brush. This will prevent the greasy matter which exudes from the scalp accumulating and forming a dry black crust, disgusting in its appearance and liable to occasion ulcerations of the skin beneath it of a most obstinate and painful character.

"Many parents are opposed to frequently washing the head of an infant, from a supposition that it will render it liable to take cold, and be otherwise prejudicial to health; no such fears, however, need be entertained; the child's health will be much more endangered by neglecting to keep its head scrupulously clean than by the too frequent application to it of water of a proper temperature. A soft brush should always be used instead of a comb; for cleansing and smoothing the hair of young children, there being less danger of the brush scratching or unduly irritating the skin."

The diet of the child is a matter of the last importance. If the mother can nurse it, her milk is not only the proper food for it, but its only suitable food. Children may be raised by hand, but to say nothing of the trouble of thus rearing them. nothing of the sickness thus induced, the mortality among infants where this system prevails, is enough to rebuke the folly and cruelty of mothers who send away their offspring to be nurtured by strangers. Dr. Condie might have multiplied his statistics on this head, but in the subjoined paragraph he has given enough:

"The difficulty of rearing an infant, when from any cause it is deprived of the maternal breast or that of a healthy nurse, is pointed out by almost every writer that has treated on the subject of infantile hygiene. In the asylums for foundlings and young infants, where feeding by the hand has been substituted for the natural nourishment, the mortality has been invariably the most appalling. Forty, fifty, sixty, and even as high as eighty and ninety per cent. of the infants being destroyed. (*Annales d'Hygiene*, t.xix.) It is true, as Auvity properly remarks, that in the domestic nursery, and where the utmost care and attention are bestowed at every moment upon a single infant, feeding by the hand is far less destructive to life. Under such circumstances, robust and healthy children have certainly been reared entirely without the breast. Still the task is a difficult one, and against the few instances in which it succeeds, we must place the very many in which it entirely fails. 'I am convinced,' says Dr. Merriman, 'that the attempt to bring up children by hand proves fatal in London to at least seven out of eight of these miserable sufferers; and this happens whether the child has never taken the breast, or having been suckled for three or four weeks only, is then weaned. In the country, the mortality among dry-nursed children is not quite so great as in London, but it is abundantly greater than is generally imagined.' When, therefore, from any cause—and the case is one of very rare occurrence—a mother is unable to suckle her infant, the breast of a proper nurse should be substituted.

"The danger which attends every attempt to rear an infant by the hand, as it is termed, is indeed now very generally recognised by mothers; the importance, however, of confining it entirely to the breast during the first months subsequent to birth, is still far from being understood. It is too often the case that an infant is forced to partake daily of substances to the digestion of which the powers of its stomach are totally inadequate, or which are altogether unadapted to afford to it wholesome nutriment, under the absurd notion that some other food, in addition to the mother's milk, is necessary to support its strength and promote its growth. The beneficial effects of the natural food are in this manner, in a great measure, counteracted, while the infant is subjected to many of the dangers resulting from dry-nursing; the functions of its digestive organs become almost invariably deranged; it suffers more or less from flatulence, griping pains, and irregularity of the bowels, and becomes weak and emaciated, even if distressing spasms of the glottis, general convulsions, or still more serious disease be not induced."

If from any cause it be absolutely necessary to feed the child, nothing is so good a substitute for the mother's breast as the milk of the cow. This is apt to become sour when kept long, especially in warm weather, and in that state disagrees with the child. We have found a great advantage in boiling the milk, by which its albumen is coagulated, and thus its tendency to ferment diminished. The effect of boiling cider in keeping it sweet is well known. At the boiling point the *ferment* in the juice of the apple is coagulated, and the saccharine principle afterwards undergoes no change. The same holds, in some degree, with milk, and the fact is one of not a little value where this article must be kept for children through a hot summer's night. The practice of adding flour to the milk, making what is called thickened milk, is bad, giving rise to acidity of the stomach and indigestion. Milk contains every element of nutrition, and all additions, unless it be a little sugar, impair its value for infants.

Our author fixes the period for weaning at a year; we are not able, however, to perceive the force of his reasons for it. He speaks of the milk as being less nutritious at the end of the year; but he does not cite the authority upon which this assertion is made. We doubt its correctness. We question whether the milk is less abundant in nutritive qualities at this advanced stage of lactation than in the first months; but even if this be true, before this time, generally, the child has learned to add many things to its bill of fare. We give the words of the author:

"The proper period for the infant to be taken from the breast, or weaned, and nourished altogether independent of the mother's milk, may be stated as a general rule to be at the termination of a year; within which period, however, the occasional use of other food may with propriety be allowed, according to the directions already given.

"At the termination of the twelfth month, the cutting of the first set of teeth is in general considerably advanced, and the digestive organs of the child are in a condition to effect promptly the solution of almost every species of plain and wholesome food; hence the propriety of depriving it entirely of the breast at this period. But when, as is occasionally the case, the process of dentition is more rapid, and the infant is at the same time healthy and vigorous, it may be weaned at any period after the tenth month; while on the other hand, when dentition is more tardy, or the infant is weak and sickly, it may be prudent, if the mother have a sufficient supply of milk, to continue it at the breast somewhat longer than a year. We are to recollect that while the health of an infant is very generally impaired by its being too early weaned, on the other hand, by confining it too long to the breast, it may likewise be seriously injured, as well from a diminution in the nutritive properties of the mother's milk, as by this being no longer adapted to the condition of the digestive organs of the infant—the functions of which have become more developed—nor to the perfect nutrition of the several organs of the body.

"In deciding upon the proper period for weaning, the season of the year should be taken into consideration. In many parts of the United States, especially in the larger cities, this is indispensable to the safety of the infant. When deprived of the breast at the commencement of or during the summer months, its liability to a disordered state of the bowels, or to a severe attack of cholera infantum, is invariably much increased. Hence the spring or autum should if possible be invariably made choice of for the period of weaning; and only under circumstances of the most imperious necessity should it ever be attempted during the season of greatest heat, unless the stomach of the infant has already become fully accustomed to other species of food, and the functions of the digestive organs are performed with perfect ease and regularity. In almost every instance, therefore, when upon the approach of summer, a mother ascertains that, from any cause, she will be unable to suckle her infant until cool weather again returns, it will be more judicious to wean it somewhat earlier than under other circumstances would be desirable; so that its stomach may become accustomed to the new kind of food which is to take the place of the mother's milk, before the intense and continued heat of the weather shall have augmented the irritability of the entire alimentary canal, and rendered this liable to disease upon a change of diet. When, however, nothing occurs to prohibit it, the infant's safety from disease will more certainly be secured, if the period of weaning be postponed until after the heat of summer is over."

We are glad to find that Dr. Condie allows children to indulge in fruit—that greatest of luxuries at that time of life. It is a most groundless and cruel prejudice which would deprive them of so high a gratification. An appetite so universal, so strong, so natural, cannot mislead; let the fruit be ripe and the child in good health, and no mischief can result from its indulgence. We go farther than Dr. C., and would allow the urchins to eat strawberries, and especially blackberries, *ad libitum.* Notwithstanding their "small, hard, insoluble seeds," we insist that they are the fruits which children can eat with the greatest impunity. The pious Dr. Butler was in the habit of saying, that "doubtless God could have made a better berry than the strawberry, but doubtless God never did;" and we agree with the learned divine. The strawberry comes first in the spring, and it were cruel to deprive the little innocents of their share of such a luxury. But the blackberry—it is a proverb of healthfulness. Who was ever known to be hurt by eating blackberries ? The following are the remarks of our author respecting fruits:

" Much difference of opinion exists as to the propriety of allowing children to partake of fresh fruits; by many they are entirely interdicted, while by others their allowance is qualified by numerous restrictions, not very easy, under ordinary circumstances, to be observed. By a healhty child, nearly all the saccharine fruits, when perfectly ripe and mellow, may be eaten in moderation with perfect safety; they furnish a very grateful and proper addition to their ordinary food. It must be recollected, however, that the wholesomeness of fruit is always in proportion to its pulpness and to its saccharine and mucilaginous qualities. Unripe fruit of every species is decidedly injurious, as well on account of its being almost entirely indigestible, as from the injury inflicted upon the stomach by the crude acid juices it contains. All fruits that are invested with a firm cuticle or skin, are also improper for children; but the ripe pulp of those fruits, as in the case of grapes, where it can be entirely freed from the skin and seeds, is in general sufficiently wholesome and nutritive. Fruit containing an abundance of small, hard and insoluble seeds, as strawberries, blackberries, whortleberries, currants, and the like, should be eaten by children only in very moderate quantities, as the seeds are liable to be retained in the alimentary canal, and may excite considerable irritation of its lining membrane.

" Cherries are among the most pernicious fruits in common use, and ought to be wholly excluded from the list of articles with which children may be occasionally indulged. Even when eaten without the stones, they are peculiarly apt to derange the bowels; and when swallowed with the stones, which with children is not unfrequently the case, they are capable of producing violent and even fatal impressions on the alimentary tube. No small number of instances have come under my notice, Dr. Eberle remarks, where the most alarming, and, in a few cases, fatal consequences resulted from the irritation of cherry-stones lodged in the bowels. Convulsions, inflammation, unconquerable constipation, and exhausting and harassing diarrhœa, are among the affections which are apt to arise from this cause.

" Fruits stewed or roasted, with the addition of sugar, are always very acceptable to children, and when tolerably ripe and not too sour, in general agree well with their digestive organs. The same remark will apply to fruits preserved in sugar; when eaten in moderation, with milk or bread, they form an innocent, and, in the case of the habitually costive, an advantageous addition to their meals. They are improper only when the bowels are peculiarly irritable and liable to be disordered from slight causes, when taken in immoderate quantities, or upon a loaded stomach. Dried fruits, when deprived of their skins, and stewed with the addition of sugar, seldom produce any injury to the stomach of a child; but when uncooked, and eaten with their skins, they are difficult of digestion, and disorder, often to a very considerable extent, the digestive organs. We have in repeated instances seen attacks of violent vomiting and purging, distressing colic, and even convulsions of a very dangerous character, produced from the use of this kind of fruit, notwithstanding the amount taken has not been in the least degree immoderate."

The subject of exercise is one of so much importance that we make long extracts from our author on this head. Left to themselves, children are sure to take exercise enough after they begin to run about, but how to manage them in helpless infancy is a point upon which Dr. C. gives the following useful instructions:

"For the first few weeks of its existence, the infant requires but little exercise of any kind; and it is not until some months have elapsed, that its organization has acquired a sufficient degree of firmness to permit of its enjoying any other than such as is of the most gentle, passive kind. From an early period, however, gentle friction with the hand over the whole surface of the body, carrying it about in a horizontal or slightly reclined position on the nurse's arms, either within doors, or, when the weather will permit, in the open air, will not only communicate a pleasurable sensation to the infant, but by promoting a free and equal circulation of the blood, contribute to the full and regular development of every part of its body. But while this amount of exercise is decidedly beneficial, and should not be neglected, we are to recollect that every species of dandling and tossing—every kind of motion, in fact, excepting of the gentlest kind, is liable to produce present discomfort, if not permanent injury.

"For the first month or two, the infant should be handled as little as possible, and never placed in an erect or sitting posture. To set it upon the lap while its bones are soft and pliable, its joints slight and imperfectly developed, and its muscles small and feeble, and to jolt it thus by the hour—its poor little head moving about as if palsied, and its tender back bending and twisting from side to side—is the very worst plan that can be adopted to minister to its comfort, or to promote its regular and symmetrical growth.

"Many nurses would appear to think that the greater the amount of motion to which an infant is subjected the better. They toss and roll it about as if it were a bundle of rags, and pull and twist its limbs till the joints crack. Under such treatment, it is not to be wondered at that the poor child moans, cries, or screams, during the whole time occupied in dressing it, or that it should suffer from deformities and other injuries.

"Another barbarous practice is that of tossing an infant up and down in the arms, held at full length from the body. The motion thus communicated is of too violent a kind to be borne with impunity by the tender frame of a young infant, to say nothing of the serious accidents which may result from the practice, even when the utmost care is observed.

"Until an infant is able to sit alone, it may be allowed to amuse itself upon its cot, or on a soft cushion spread upon the floor; here it will lie for hours, until it acquires strength to roll about, and is much more contented, and thrives far better, than when continually nursed in the arms or upon the lap; the danger of deformity is likewise avoided, and the child acquires at a more early period the power to raise itself and sit upright without assistance; and at nine or ten months will, in general, be able to get upon its feet and walk.

"When sufficiently old to be attracted by surrounding objects, carrying it frequently into the open air, especially in the country, during the mildest seasons of the year, has a highly beneficial tendency. The freshness, beauty and variety of the scenes of nature are highly attractive, even at a very early period of life; and independently of the healthy influence of a pure and unconfined atmosphere, the impressions thus resulting are always of a decidedly salutary kind.

In carrying an infant, some important precautions are necessary. The spine, being soft and yielding, is incapable of supporting the weight of the head and other parts of the body which rest upon it in the erect posture. To prevent deformity, therefore, an infant should not be held seated upon the arm of its nurse or attendant; it ought always to be carried in an inclined position, so that the head, and every part which bears upon the spine, may receive an equal and adequate support. Neither should the child be carried for too long a time in the nurse's arms without changing the position in which it is held; otherwise there is danger of its becoming permanently deformed, from its body being twisted to one or the other side. To obviate this, the child should be carried alternately on both arms. Even in suckling an infant, it is important that it be not confined exclusively to one breast, but nourished alternately from both, as well to prevent its contracting a crookedness of form, as to guard against its acquiring a habit of squinting, from one of its eyes, while nursing, being constantly directed towards one point.

"Infants in general derive much pleasure from riding in a little carriage drawn by the hand; and as it affords a convenient means of conveying them frequently, when the weather is mild and otherwise favorable, to one of the public squares or parks which exist in most of our larger cities, or even to a garden or field without the town, where they may enjoy a free and fresh air, this species of exercise may be occasionally substituted for carrying in the arms—to which, for out-door excursions, it is, for very young infants, in many points of view preferable.

"The body of the carriage should be long enough to permit the infant to lie down at full length, and the sides ought to be sufficiently high to prevent it falling or rolling out. The wheels should be low, in order to lessen the liability of oversetting, and they should be carefully secured against running off when the carriage is in motion. Very young infants should be laid down in the carriage on a pillow or soft mattress, with the head slightly elevated, and so confined at the sides as to prevent them from rolling when in motion. After the child has acquired some degree of strength, it should be placed in a semirecumbent posture, with its head and back well supported by pillows, etc.; and when it is capable of supporting its head, it may be permitted to sit upright, and properly secured against being thrown from side to side by the rolling of the carriage.

"The carriage should be drawn by none but a prudent and trusty person, with a moderate and steady motion, and never over rough or uneven ground; when the motion is too rapid, or uneven and jolting, much injury may be inflicted on the infant.

"Children, when placed in the carriage, should never be kept standing in the sun, if it be warm; nor should they be kept motionless when the weather is cool. In cold weather, they should be amply covered, since from the passiveness of their situation, they will require additional clothing. Nor should the ride ever exceed half an hour at a time, especially if the child is observed to become sleepy; it being hazardous to allow children to sleep in the open air; and after a few trials, the exercise almost invariably will predispose them to sleep, and thus destroy the benefit of the exercise, as well as interfere with their regular habits of repose.—(*Dewees.*)

"It is only towards the end of the ninth or tenth month, and, when the child is feeble, even much later, that it is proper to teach it the use of its feet. It is perhaps always better that before an infant attempts to walk, it should first learn to crawl. With this intention, it should be placed upon a soft carpet, where it soon busily employs itself, moves and extends its limbs, or rolls about to reach its playthings. The instinctive desire for locomotion, and the various things that attract it in different parts of the room, will soon induce it to crawl upon its hands and feet. This it should be allowed and even encouraged to do; as children who are permitted this 'useful intermediate muscular discipline,' are sure to acquire a much firmer step, and enjoy more robust health, than those who have been taught to walk without it.

"In no instance should any particular attempt be made to induce children to use their feet at an early period, nor until the bones have acquired a sufficient degree of solidity to sustain the body, without the danger of their becoming bent by its weight.

"In teaching a child to walk, it should be left pretty much to its own efforts; all artificial support is injurious. As generally applied, such support has a tendency to produce an unnatural elevation of the shoulders, while the infant, depending upon it almost alone for maintaining the upright position of its body, is accustomed to bend too much forward or to one side. By this may be laid the foundation of a permanent deformity, or at least of an ungraceful gait, which it is impossible in after life to correct.

"While in the nursery, infants may be taught to rise from the floor by laying hold of chairs; and if occasionally supported under the arms, they will easily learn to stand erect; but they should never be raised up by one arm only. At an early age they may be held under both arms; and when thus supported, if the hands of the attendant be occasionally withdrawn for a moment, they will soon acquire the power of standing alone. Mild and persuasive language ought to be used in these experiments; while the infant may be encouraged by some toys placed at a little distance, which will induce it to stretch out its little arms, and endeavor to advance towards the place containing the desired objects. By such means it may be allured to visit different parts of the room. The first journey of this kind ought to be attempted only from one neighboring chair to another; afterwards the little traveler may run towards its mother or nurse, who with extended arms stoops to receive it. As the infant improves in its efforts to walk alone, the chairs may be placed at a greater distance from each other, until finally it walks firmly in every direction, without assistance of any kind.—(*Struve.*)"

We have examined only a few of the introductory chapters of this work, but it is not our intention to extend our notice to the main body of it, in which the diseases of infancy and childhood are discussed. For this we must refer the reader to the work itself, and in doing so we feel assured that we are referring to one of the richest sources of instruction on the subject in our language. Taken as a whole, in our judgment, Dr. Condie's treatise is the one from the perusal of which the practitioner in this country will rise with the greatest satisfaction. Defects, doubtless he will discover, but they will appear small in comparison with the learning and sound judgment displayed on all its pages.

